# Predictors of treatment failure and time to detection and switching in HIV-infected Ethiopian children receiving first line anti-retroviral therapy

**DOI:** 10.1186/1471-2334-12-197

**Published:** 2012-08-24

**Authors:** Tigist Bacha, Birkneh Tilahun, Alemayehu Worku

**Affiliations:** 1 Department of Pediatrics and Child Health, Addis Ababa University, Addis Ababa, Ethiopia; 2 School of Public Health, Addis Ababa University, Addis Ababa, Ethiopia

**Keywords:** HIV/AIDS, Immunological, Clinical, Predictors, Treatment failure, Delay, Switching, Ethiopia

## Abstract

**Background:**

The emergence of resistance to first line antiretroviral therapy (ART) regimen leads to the need for more expensive and less tolerable second line drugs. Hence, it is essential to identify and address factors associated with an increased probability of first line ART regimen failure. The objective of this article is to report on the predictors of first line ART regimen failure, the detection rate of ART regime failure, and the delay in switching to second line ART drugs.

**Methods:**

A retrospective cohort study was conducted from 2005 to 2011. All HIV infected children under the age of 15 who took first line ART for at least six months at the four major hospitals of Addis Ababa, Ethiopia were included. Data were collected, entered and analyzed using Epi info/ENA version 3.5.1 and SPSS version 16. The Cox proportional-hazard model was used to assess the predictors of first line ART failure.

**Results:**

Data of 1186 children were analyzed. Five hundred seventy seven (48.8%) were males with a mean age of 6.22 (SD = 3.10) years. Of the 167(14.1%) children who had treatment failure, 70 (5.9%) had only clinical failure, 79 (6.7%) had only immunologic failure, and 18 (1.5%) had both clinical and immunologic failure. Patients who had height for age in the third percentile or less at initiation of ART were found to have higher probability of ART treatment failure [Adjusted Hazard Ratio (AHR), 3.25 95% CI, 1.00-10.58]. Patients who were less than three years old [AHR, 1.85 95% CI, 1.24-2.76], chronic diarrhea after initiation of antiretroviral treatment [AHR, 3.44 95% CI, 1.37-8.62], ART drug substitution [AHR, 1.70 95% CI, 1.05-2.73] and base line CD_4_ count below 50 cells/mm3 [AHR, 2.30 95% CI, 1.28-4.14] were also found to be at higher risk of treatment failure. Of all the 167 first line ART failure cases, only 24 (14.4%) were switched to second line ART with a mean delay of 24 (SD = 11.67) months. The remaining 143 (85.6%) cases were diagnosed to have treatment failure retrospectively by the authors based on their records. Hence, they were not detected and these patients were not offered second line ARTs.

**Conclusions:**

Having chronic malnutrition, low CD_4_ at base line, chronic diarrhea after initiation of first line ART, substitution of ART drugs and age less than 3 years old were found to be independent predictors of first line ART failure in children. Most of the first line ART failure cases were not detected early and those that were detected were not switched to second line drugs in a timely fashion. Children with the above risk factors should be closely monitored for a timely switch to second line highly active anti-retroviral therapy.

## Background

The human immunodeficiency virus (HIV) epidemic continues to be a major challenge to global health. According to the 2009 UNAIDS report, about 33.4 million people worldwide were living with HIV/AIDS (LWHA) and of them 2.1 million were children. In 2008 alone, 430 thousand children under the age of 15 were newly infected with HIV, and 280 thousand children lost their lives because of acquired immune deficiency syndrome (AIDS) [1]. Sub-Saharan Africa continues to be the region most affected by the pandemic, with nearly 90% of all new childhood HIV infections and approximately 7 of 10 recorded deaths occurring in this region [1,2].

In 2006, Ethiopia reported that there were nearly 134 thousand children under the age of 15 years old LWHA [3]. In 2009, there were 29, 546 pediatric patients “ever enrolled in HIV care”, and 13,650 were “ever started on anti-retroviral therapy (ART)” [4]. In 2009, there were 10,361 children who were on first line ART regimens and 135 children on second line ART regimens [4].

Many studies have reported on the success of highly active anti retroviral therapy (HAART) in improving clinical and immunologic outcomes of children [5-9]. However, as the use of HAART increases, the issue of drug resistance and subsequent treatment failure presenting as one or more of clinical, immunological or virological ART failure appears as a challenge [10-12].

Clinical failure is defined as the occurrence of new opportunistic infections (OIs) or malignancies; failure to sustain growth rate in a child who had showed an initial response; or loss of neurodevelopment milestones excluding cases of inadequate nutrition or tuberculosis [10-12]. Immunologic failure is a rapid, serial rate of decline to the cut-off values for severe immunodeficiency state for age or below; or a fall in CD_4_ by 50% from the recorded peak in the absence of a concurrent illness [10,12]. Generally, treatment failure is different from immune reconstitution inflammatory syndrome (IRIS). IRIS is a paradoxical clinical deterioration that occurs even when the immune system begins to improve, while no immunologic improvement occurs in treatment failure [10,13].

Factors associated with ART failure may include socio-demographic factors (e.g. age, gender, being an orphan), baseline clinical factors (e.g. high pre-treatment viral load, low pre-treatment CD_4_ count, prior World Health Organization (WHO) stage), drug-drug interactions (between the ART and concomitantly administered drugs), drug side-effects, drug toxicity or inadequate adherence to treatment [14-22].

In an Ethiopian university hospital, among children on first line ART, clinical treatment failure and immunologic treatment failure were diagnosed in 6.2% and 11.5% respectively [22]. The presence of chronic gastroenteritis or the appearance of a new opportunistic infection after starting treatment was associated with immunologic treatment failure [22]. Deaths that occurred within the first three months of therapy were associated with higher rates of default, treatment failure, severe drug toxicity and regimen changes [22].

ART failure is not a common diagnosis in most centers in Ethiopia. Very few patients among the needy are started on second line ART regimens. Previous studies have evaluated the patterns associated with switching from first line ART regimes to second line ART regimes; however, studies that evaluate the factors associated with first line ART regime failures in limited resource settings like Ethiopia and Africa at large are scarce. The objective of this article is to report on the predictors of first line ART regime failure, the detection rate of ART regime failure, and the delay in switching to second line ART drugs.

## Methods

### Setting

We conducted a retrospective cohort study of children with HIV/AIDS in Addis Ababa, the capital of Ethiopia. The city area is 450 square kilometers with an estimated population of 2.7 million. The four major public pediatric referral hospitals in the city with strong pediatric HIV/AIDS care and treatment centers were selected for the study, and they include Tikur Anbessa, Yekatit 12, Zewditu Memorial and ALERT hospital. All of the children at the four ART centers who had been started on first line ART drugs based on the national guideline by professionals working at the respective center were included. Ethical approval was obtained from the institutional review board of the Addis Ababa health Beuro and each hospital in the study. Confidentiality was assured by making the questionnaires anonymous [10].

### Sample

We used retrospective data of 1186 children under 15 years old who were on ART regimes between March 2005 and March 2011 at the ART centers of the four major hospitals. Children seen at these centers were followed based on the national guidelines which recommends that children are evaluated two weeks after initiation of ART, every month for the next two months, and every three months afterwards. Children who had a minimum of two follow-up visits with at least one visit six months post initiation of ART were included in the sample. Children who took ART only for prevention of mother to child transmission (PMTCT) were excluded.

### Data/measures

We abstracted data from the patients’ medical records to a structured data retrieval form. These records included: clients’ age, gender, weight, height, duration of follow up, clinical and laboratory data (WHO stage, opportunistic infections, CD4 count and white cell count), the ART regimen and prophylaxis (Co-trimoxazole or Isoniazid), and adherence were all recorded on a structured data retrieval form. The data were collected from the records starting from the time of ART initiation and every six month afterwards. Because growth was not routinely plotted on the growth chart by the professionals working at the centers, we used the recorded weight and height to plot growth for each child every six months.

We used the standard definition of clinical failure defined as occurrences of new opportunistic infections (OIs) or malignancies or failure to sustain growth rate in a child who had showed response initially or loss of neurodevelopmental milestones excluding other causes including inadequate nutrition and tuberculosis [10-12]. Growth failure was diagnosed when a child’s growth trajectory (weight-for-age, height for age and/or weight for height) decreased by two percentile curves or when it was below the third percentile for age in the standardized curves of WHO. In the current study, growth failure was taken as a sign of ART failure when the altered growth stayed for more than six months at follow up. Immunologic failure was defined at follow up according to the national guideline. Virologic criteria were not used because of the complexity of defining ART failure in children using viral load and the unavailability of regular virologic tests in Ethiopia [10]. Patients with opportunistic infections were differentiated from those patients having IRS based on the pattern of their CD_4_ counts [10].

In this cohort, the time to detection of treatment failure indicates the time between ART initiation and detection of failure of first line ART (clinical or immunologic failure). Time to switch to second line ART regimens represents the time between detection of first line treatment failure and the time of initiation of second line drugs. Adherence was defined as optimal if pill count or self report was greater than or equal to 95% and sub optimal if pill count or self report was less than 95%. IRIS was noted when opportunistic infections occurred during the first several months of initiation of ART in the presence of immune recovery [10,13].

### Statistical analysis

The data were entered and analyzed using SPSS version 16. Z-scores and percentiles of anthropometric values were calculated using Epi-Info/ENA version 3.5.1 (CDC, Atlanta, Georgia, and USA, 2008) Descriptive statistics including counts, percentages, medians, ranges, means, and standard deviations were calculated. Association between the outcome and the independent variables was taken as significant at P < 0.05. First-line ART failure was further described by subcategories of demographic and clinical characteristics. Independent variables that were significantly associated with first-line ART failure in bi-variate analysis were further examined in multivariate analysis. The Cox proportional-hazards model was used to assess the predictors associated with treatment failure. Those factors with P < 0.2 in the bi-variate model were included in the multivariate analysis. Kaplan-Meier survival methods were used to estimate the probability of treatment failure. Log rank test was employed to compare survival curves.

## Results

From the 1,186 children on first line HAART, 608 (51.2%) were female (Table 1). Four hundred four (34.1%), 315 (26.6%), 252 (21.2%) and 215(18.1%) had a follow up at Tikur Anbessa hospital, Zewditu hospital, Yekatit 12 hospital and ALERT hospital respectively. The mean age at initiation of ART was 6.22 (SD = 3.10) years and ranged from 2- to 14 years old. The mean follow up period after ART initiation in the current study was 37 ± 17 months, with minimum 6 months to maximum of 74 months.

**Table 1:** **Baseline characteristics of children on HAART**^**€ **^**Treated in four ART**^**# **^**Centers in Addis Ababa, Ethiopia followed from 2005–2011**

**Characteristic**	**Number (%)**
Age	
-≤ 11 months	46(3.9 )
-11 to 34	169 (14.2)
-34 to 59	212 (17.9)
-≥60 month	759 (64)
Sex	
-Female	608 (51.2%)
-Male	578 (48.8%)
Duration of Follow up	
-Less than one year	265(22.3)
-One to two years	270(22.8)
-Two to three years	248(20.9)
-Three to four years	225(19.0)
-More than four years	178(15.0)
Parental status	
-Both alive	427(36.96)
-Either dead	468(40.51)
-Both dead	260(22.5)
-Unknown	31 (2.6)
Serology of the care takers	
-Positive	773(65.2)
-Negative	55(4.6)
-Unknown	358(30.2)
Primary care taker	
-Both parents	415(35)
-Mother	249(21)
-Father	156(13.2)
-Relatives	125(10.5)
-Guardians /neighbors	142(12)
-Orphange	99(8.3)
WHO stage	
-I	42(3.5)
-II	305(25.7)
-III	626(52.8)
-IV	213(18)
Weight –for- Age	
-Below 3^rd^ centile	172(14.5)
->3^rd^ centile < 97^th^ centile	192(16.2)
-Above 97^th^ centile	44(3.7)
-Unknown	778 (65.6)
Height- for- Age	
-Below 3^rd^ centile	327(27.6)
->3^rd^ centile < 97^th^ centile	763(6.3)
-Above 97^th^ centile	81(6.8)
-Unknown	15(1.3)
Weight- for –Height	
-Below 3^rd^ centile	105(8.9)
->3^rd^ centile < 97^th^ centile	245(20.7)
-Above 97^th^ centile	707(59.6)
-Unknown	129(10.9)
Regimen started at initiation of ART	
-4a (D4T + 3TC + NVP)	388(32.7)
-4b(D4T + 3TC + EFV)	106(8.9)
-4c(ZDV + 3TC + NVP)	300(25.3)
-4d(ZDV + 3TC + EFV)	361(30.4)
Infant ART prophylaxis	
-NVP	9(0.8)
-NVP + ZDV	977(82.4)
-None	977(82.4)
-Unknown	181(15.3)
Immunosupression at initiation of ART $	
-Not Significant	160(13.5)
-Mild Immunosupression	145(12.2)
-Advanced Immunosupression	331(27.9)
-Severe Immunosupression	450(37.9)
-Unknown	100(8.4)
Chronic Diarrhea after initiation	
-Yes	16 (1.3)
-None	1170(98.7)

€ Highly Active Antiretroviral Therapy # Antiretroviral Therapy $ Level of immunosupression is based on the Ethiopian National ART guideline [10].

PMTCT service was very low; only 2.4% of the children received the service (0.8% received only Nevirapine (NVP) and 1.6% received NVP and Zidovudin (ZDV). ART initiation occurred due to advanced World Health Organization (WHO) clinical staging in 210 (17.7%) patients, low CD_4_ count in 248 (20.9%) patients and both WHO and immunologic criteria in 719 (60.6%) patients. Infants with diagnosed HIV/AIDS accounted for 9 (0.8%) of the patients which is a criteria for initiation by itself. The regimen type initiated at base line was NVP based in 688 (58%) patients and Efavirenz (EFV) based in 467 (39.3%) patients (Table 2).

**Table 2:** **Sociodemographics as predictors of first line ART failure in children with HIV/AIDS**^**¥ **^**Treated in Four ART**^**3**^**Centers in Addis Ababa, Ethiopia followed from 2005-2011**

**Covariate**	**Treatment failure**	**Treatment success**	Crude hazard risk (95 CI**)	Adjusted hazard risk (95 CI**)
Age				
-< 36 months	46(20.8)	175(79.2)	1.87(1.26-2.76)	1.85(1.24-2.76)
-36-60 months	28(13.3)	183(86.7)	1.09(0.69-1.71)	1.09(0.69-1.72)
->60 months	93(12.3)	661(87.7)	1	1
Gender				
-Female	96(15.8)	512(84.2)	1.34(0.96-1.86)	1.30(0.91-1.87)
-Male	71(12.3)	506(87.7)	1	1
Primary Care Taker				
-Parent (single/both)	119(14.5)	701(85.5)	1.13(0.78-1.61)	
-Non-parent	48(13)	318(87)	1	
Care taker HIV sero-status				
-Reactive	112(14.5)	661(85.5)	1.12(0.78-1.62)	
-Unknown	47(13.1)	311(86.9)	1.13(0.50-2.53)	
-Negative	8(14.5)	47(85.5)	1	
PMTCT Service				
-Present	3(12.5)	21(87.5)	0.93(0.27-3.25)	
-None	119(14.4)	705(85.6)	1.10(0.76-1.59)	
-Unknown	45(13.3)	293(86.7)	1	
Infant ART prophylaxis				
-Yes	5(18)	23(82)	1.97(0.67-5.81)	1.43(0.47-4.31)
-None	144(14.7)	833(85.3)	1.57(0.93-2.63)	1.56(0.93-2.63)
-Unknown	18(10)	163(90)	1	1
Disclosure^£^				
-Disclosed	41(13.2)	270(86.8)	1.04(0.69-1.57)	
Not disclosed	73(12.7)	501(87.3)	1	

**Confidence interval £ Is telling children about their HIV status and was done for children older than 8 years ¥ Human Immunodeficiency virus/ Acquired Immunodeficiency syndrome ^3^ Antiretroviral therapy.

A large number of children were malnourished at base line with 14.5%, 27.9% and 2.4% of children falling at or below the third percentile for weight for age, height for age and weight for height respectively. At ART initiation, 450 (37.9%) of the children were found to be in severe age adjusted immunodeficiency state based on CD_4_ percentage for those less than 5 years of age or CD4 count for those above 5 years of age. The CD_4_ count was less than 50 cells/mm3 in 80 (6.7%) of all children. The mean CD_4_ count for the whole cohort at initiation of ART was 300 cells/mm3 (SD = 271 cells/mm3). Though a large number of children had severe immunodeficiency at base line, the mean increment of CD_4_ count was remarkable until 12 months after initiation of ART (P < 0.0001). CD_4_ count continued to increase slowly until 36 months after initiation of ART though not statistically significant (P = 0.48). CD_4_ count started to decline after 42 months of initiation of ART. The mean CD_4_ count was 685 cells/mm3 (SD = 560cells/mm3), 741 cells/mm3 (SD = 373 cells/mm3), 772 cells/mm3 (SD = 423 cells/mm3) and 580 cells/mm3 (SD = 421cells/mm3) at one year, two years, three years and five years after initiation of ART respectively.

Forty one (3.5%) children had opportunistic infections after the initiation of ART; the mean time for onset of opportunistic infections after initiation of ART was 20.8 ±15.9 months with a range of 2–62 months. The most common opportunistic infections were disseminated tuberculosis (TB) (3%), *pneumocystis carinii* pneumonia (PCP) (0.2%), recurrent intractable oral thrush (0.2%), and recurrent severe pneumonia (0.1%). The overall adherence rate was reported to be optimal; with no episode of suboptimal adherence in 98.4% and two or more episodes of suboptimal adherence in only 1.6% of the patients.

Anti-retroviral drug substitution occurred for 105 (8.9%) patients. Substitution was necessary because of toxicity for 94 (7.9%) patients, and concomitant TB treatment for 7(0.6%) patients. Toxicity was more common in the 4c (ZDV + 3TC + NVP) regimen. Among the 94 children with toxicity: 33 (35.1%), 29 (31.0%), 20 (21.3%) and 12(12.6%) were on 4c (ZDV + 3TC + NVP), 4a (D4T + 3TC + NVP), 4b (D4T + 3TC + EFV) and 4d (ZDV + 3TC + EFV) regimens respectively. The most common recorded side effects of drug toxicity was anemia; 21(22.3%), followed by skin rash; 13 (13.8%), hepatotoxicity; 10/ (10.6%), lipodystrophy; 6 (6.4%), neuropathy; 4 (4.3%), night terror; 1(1.1%), and severe nausea 1 (1.1%).

Patients who had height for age less than the 3^rd^ percentile at initiation of HAART were found to have a higher probability of treatment failure [AHR, 3.25 95% CI, 1.00-10.58] (Table 3). Compared to patients with base line CD_4_ count above 50 cells/mm3, those below 50 cells/mm3 had higher probability of treatment failure [AHR, 2.30 95% CI, 1.28-4.14]. Similarly the presence of chronic diarrhea after initiation of ART [AHR, 3.44 95% CI, 1.37-8.62] and ART drug substitution [AHR, 1.70 95% CI, 1.05-2.73] were also found to be a risk of treatment failure.

**Table 3:** **Clinical and treatment data of children with HIV/AIDS **^**α **^**Treated in four ART**^**¥ **^**Centers in Addis Ababa, Ethiopia followed from 2005-2011**

**Covariate**	**Treatment failure (%)**	**Treatment success (%)**	Crude hazard risk (95 CI**)	Adjusted hazard risk (95 CI**)
Weight for Age				
-Below 3^rd^ centile€	52(30.2)	120(69.8)	2.74(1.09-6.89)	0.54(0.13-2.25)
>3^rd^ < 97^th^ centile	107(55.7)	85(44.3)	0.79(0.32-1.92)	0.41(0.11-1.55)
Above 97^th^ centile	6(13.6)	38(86.4)	1	1
Height for Age				
Below 3^rd^ centile	88(27)	239(63)	2.61(1.29-5.29)	3.25(1.00-10.58)
>3^rd^ < 97^th^ centile	66(8.7)	697(91.3)	0.67(0.33-1.37)	1.05(0.33-3.33)
Above 97^th^ centile	10(22.2)	71(77.8)	1	1
Weight for Height				
Below 3^rd^ centile	15(14.3)	90(85.7)	1.10(0.61-1.98)	
>3^rd^ < 97^th^ centile	41(16.7)	204(83.3)	1.33(0.89-1.98)	
Above 97^th^ centile	93(13.2)	614(86.8)	1	
WHO Clinical stage				
Stages 1 or 2	58(12.7)	400(87.3)	0.84(0.59-1.18)	
Stage 3 or 4	107(14.8)	617(85.2)	1	
Adherence to HAART				
Optimal	162(14)	1006(86)	0.41(0.15-1.19)	
Suboptimal	5(27.3)	13(72.7)	1	
Initial HAART Regimen				
PI based ^£^	10(32.3)	21(67.7)	3.03(1.40-6.55)	1.05(0.33-3.89)
NNRTI + NRTI	157(13.6)	998(86.4)	1	1
HAART drug substitution				
Yes	29(23.6)	94(76.4)	2.07(1.31-3.25)	1.695(1.053-2.728)
None	138(13)	925(87)	1	1
HAART Dosing preparation				
Fixed	9(17.6)	42(82.4)	1.18(0.56-2.49)	
Separate	109(15.4)	600(84.6)	1	
CD_4_ Count at initiation				
(cells/mm3)				
<50	19(23.8)	61(76.2)	2.39(1.38-4.15)	2.30(1.28-4.14)
≥50	112(11.5)	859(88.5)	1	1
Chronic Diarrhea after initiation of ART^¥^				
Yes	6(37.5)	10 (62.5)	3.76(1.35-1.50)	3.44 (1.37-8.62)
None	161(13.8)	1009(86.2)	1	1

**Confidence interval £-Started for infants who took prophylaxis; α Human Immunodeficiency Virus/Acquired Immunodeficiency Syndrome ¥ Antiretroviral therapy € Percentile.

There were 167 (14.1%) children with HIV/AIDS who had evidence of first line ART failure of which 70 (5.9%) had clinical treatment failure, 79(6.7%) immunologic failure and 18 (1.5%) developed both immunologic and clinical failure. Out of all children with first line ART failure, only 24 (14.4%) were identified at the four ART centers. The mean time of detection of treatment failure was 19.7 months (SD = 14 months) and the mean time to switch to second line ART regimen, for those switched, was 24 months (SD = 11.67 months).

## Discussion

The current study found that in patients with treatment failure, clinical failure was the most common followed by immunologic failure with only a small proportion having both clinical and immunologic failure. There was a striking delay in the detection of ART failure. Only 24 (14.4%) patients were labeled as having treatment failure in all the ART centers and were switched to second line therapy. Our finding of 167 first line ART failures in the four hospitals is greater than the 135 pediatric patients that were identified by the national report and put on second line drugs following first line ART failure [3,4]. Our findings demonstrates that a significant number of pediatric treatments failures are not identified and/or not switched to second line ART.

The current study shows that children under three years old, chronic malnutrition, low CD_4_ count at ART initiation, chronic diarrhea after ART initiation and ART drug substitution are independent predictors of first line ART failure. Similarly, studies elsewhere showed that younger age is a predictor of virologic failure [21,23-25]. Chronic malnutrition was also shown to be a predictor of treatment failure previously in Ethiopian and Singapore [22,25]. Likewise, an Ethiopian study in Jimma showed that chronic diarrhea before initiation of ART was predictive of treatment failure [22]. Our finding of a higher risk of first line ART failure in children who had ART drug substitution in the current study is consistent with a study conducted in Malawi [17].

Some possible mechanisms by which the above predictors could affect treatment include difficulty of drug administration in those below three years of age, the high prevalence of underlying malnutrition which increases the risk of infection, and chronic diarrhea which in turn decrease drug absorption and impair metabolism. The children with low CD_4_ count at initiation could have an increased risk of opportunistic infections which further impairs the positive effects of the ART.

Only a few patients among those who actually had treatment failure were detected at the ART centers. The mean duration of treatment failure on first line ART and the mean duration from detection of treatment failure to switch to second line ART were longer in our study than the studies in Malawi [17] and South Africa [19]. One possible reason for the lack of timely detection of ART failure in the current study is the poor recording of growth parameters (through plotting) which was not regularly done in all the ART centers included in the study. The rate of development of OIs in the current study is less than that reported in Jimma [22]; but higher than the Cambodian [26] and the United States perinatal AIDS collaborative transmission studies [8].

Only 2.4% of the studied children received drugs for infant prophylaxis, despite the currently improving rate of institutional deliveries where PMTCT services are available. In urban areas (including where this study was conducted), the health facility delivery based on the recent national data is nearly 50% [27]. Thus, most of these HIV infections could have been prevented if these children received PMTCT services.

Adherence to ART regimens was not a statistically significant predictor of treatment failure in this study. This could be due to the high self-reported adherence rate in the current study which made comparison between the groups difficult. The adherence rate in the current study is higher than a cross-sectional study done in the same region [28] which could be explained by the retrospective nature of the current study.

The retrospective nature of this study is a limitation due to incompleteness of the records. The temporal relation between CD_4_ count determinations and concurrent illnesses was sometimes difficult to determine due to the retrospective nature of the study. Deaths, dropouts, and transfer patients were not included and this could have biased our estimates. Although children who had growth failure that persisted for 6 months were included as treatment failure cases, it was unclear whether nutritional therapy was given adequately which would have influenced existence of treatment failure. Finally, the lack of viral load to diagnose treatment failure is the other limitation.

## Conclusions

In conclusion, children under 3 years old, chronic malnutrition at ART initiation, chronic diarrhea after the start of ART, drug substitution during the course of ART, and having CD_4_ count less than 50 at base line were independent predictors of increased risk of treatment failure. The majority of patients with treatment failure were not diagnosed with treatment failure in a timely fashion, and there was a significant delay in switching to second line drugs even after treatment failure to first line drugs was identified. Hence, prevention and treatment of malnutrition in this group of children should be part of the care and treatment they receive as a part of their HIV treatment. A regular follow up of these children with growth parameters (including regular plotting of weight and height) should also be a part of the routine follow-up at all ART centers. Early initiation of second line drugs in case of first line treatment failure should occur in a more timely manner at these centers.

## Competing interests

The authors have no conflict of interest.

## Authors’ contributions

TB conceived the initial idea, developed the proposal, collected data, did the analysis and write up. BT edited the manuscript and formatted it for publication. AW advised during proposal development, edited the proposal, advised during data analysis and edited the final paper. All the authors read and approved the final manuscript.

## Pre-publication history

The pre-publication history for this paper can be accessed here:

http://www.biomedcentral.com/1471-2334/12/197/prepub
